# Alterations in Gut Microbiome Composition and Function in Irritable Bowel Syndrome and Increased Probiotic Abundance with Daily Supplementation

**DOI:** 10.1128/mSystems.01215-21

**Published:** 2021-11-02

**Authors:** Joann Phan, Divya Nair, Suneer Jain, Thibaut Montagne, Demi Valeria Flores, Andre Nguyen, Summer Dietsche, Saurabh Gombar, Philip Cotter

**Affiliations:** a Sun Genomics, Inc., San Diego, California, USA; b Atropos Health, Palo Alto, California, USA; c ResearchDx, Inc., Irvine, California, USA; Pacific Northwest National Laboratory

**Keywords:** irritable bowel syndrome, gut microbiome, metagenomics, probiotics, prebiotics, synbiotics

## Abstract

Irritable bowel syndrome (IBS) is characterized by abdominal discomfort and irregular bowel movements and stool consistency. As such, the gut microbiome has been posited as being influential for the syndrome. However, identifying microbial features associated with IBS symptom heterogeneity is difficult without large cohorts. Our aim was to identify microbial features associated with IBS and IBS subtypes compared to healthy controls and to determine if a synbiotic supplementation intervention could decrease the proportion of those microbial features. Stool samples from 490 individuals with IBS (including all dominant subtypes) and 122 individuals without IBS were analyzed with metagenomic sequencing. One hundred thirty-four IBS subjects were followed over time while receiving daily synbiotic supplementation, the composition of which varied between participants. IBS participants had significantly lower alpha diversity, an enrichment in Gram-negative bacteria, and a reduction in pathways associated with short-chain fatty acid and vitamin synthesis. *Shigella* species were significantly associated with IBS, while Eubacterium rectale and Faecalibacterium prausnitzii were associated with healthy controls. Random forest identified unique and overlapping microbial features associated with each IBS subtype. Longitudinal assessment of 134 IBS subjects receiving synbiotic supplements demonstrated a significant difference in microbial features and an increase in probiotic abundance across time. We identified microbial features that differentiate healthy and IBS subtypes. Synbiotic supplementation in IBS subjects did not result in alpha diversity change in the microbiome but did demonstrate changes in microbial features. Future work is needed to determine if the observed microbiome changes are associated with IBS symptom improvement.

**IMPORTANCE** An estimated 35 million people in the United States and 11.5% of the population globally are affected by IBS. Immunity, genetics, environment, diet, small intestinal bacterial overgrowth (SIBO), and the gut microbiome are all factors that contribute to the onset or triggers of IBS. With strong supporting evidence that the gut microbiome may influence symptoms associated with IBS, elucidating the important microbes that contribute to the symptoms and severity is important to make decisions for targeted treatment. As probiotics have become more common in treating IBS symptoms, identifying effective probiotics may help inform future studies and treatment.

## INTRODUCTION

Irritable bowel syndrome (IBS) is characterized by chronic gastrointestinal discomfort and abdominal pain with changes in bowel habits or stool consistency. IBS affects approximately 11.5% of the population, depending on the country or region (1). Because of the high prevalence of IBS, symptoms contribute to changes in quality of life and increases in health care and economic burden (2–5). There are four symptomic subtypes, IBS-C (constipation), IBS-D (diarrhea), IBS-A (alternating), and unspecified (IBS-U) (6). Individuals with IBS-A experience alternating symptoms of chronic diarrhea and constipation. The criterion for diagnosis is symptom based and codified in the Rome IV criteria; there is not yet consensus on the underlying etiology of IBS (7, 8). In addition, there are different factors that contribute to the various symptoms of IBS, including diet, immune response, host genetics, environmental stress, gut microbiome composition, and dysbiosis (9, 10).

Currently, the role of the gut microbiome in IBS symptoms and recovery remains poorly understood. A “healthy” gut microbiome may be undefined, but there are microorganisms associated with an unhealthy microbiome, including microorganisms that induce inflammation or dysbiosis that contribute to the symptoms associated with IBS. Changes in microbiome composition also impact the microbial functional potential and metabolism, which may in turn affect host physiology. For example, studies indicate individuals experiencing IBS-C show microbiome signatures such as increased Pseudomonas and Bacteroides thetaiotaomicron with depletion of *Paraprevotella* and significant associations with Fusobacterium nucleatum and Megamonas hypermegale (11). In addition, research has characterized the microbiome of subjects with IBS-C with the biosynthetic pathways for sugar and amino acid metabolism; subjects with IBS-D had microbes that predominated in the pathways for nucleotides and fatty acid acid synthesis (11). 16S rRNA amplicon sequencing studies have also described an enrichment of *Clostridiales*, *Prevotella*, and *Enterobacteriaceae*; reduced microbial richness; and the presence of methanogens in IBS (12, 13). However, amplicon studies can be subject to amplification bias, yielding variable results, and do not resolve species-level taxonomic classification. Alternatively, several studies limited by sample size and methodology have not shown a difference between a healthy cohort and individuals with IBS (14).

Because of the differences in IBS symptoms that people experience and the individual nature of the syndrome, there is no standardized treatment or dietary recommendations to alleviate IBS symptoms (15). The antibiotic rifaximin has been shown to be an effective treatment for IBS-D (16, 17). However, rifaximin is ineffective for all IBS subtypes and antibiotic usage may be associated with an increased risk for IBS (18–21). There are additional options for treatment, including pharmaceutical options and fecal transplants, but these options are not always feasible and can be invasive. The administration of live microbial organisms, in the form of probiotics, has gained popularity with patients to alleviate their symptoms. Probiotics can alter the microbiome of patients with and without IBS (22, 23), depending on their endogenous microbiome (24). Microbes not present in the current gut microbiome can also be reestablished through probiotic supplementation (24). In individuals with IBS, there is correlative depletion of *Bifidobacterium* and *Lactobacillus* (8). Therefore, reintroducing these microbes as probiotics into the gut of individuals with IBS may lead to phenotypic changes and reduction of IBS symptoms, as demonstrated by clinical trials (25, 26). Treatment of subjects with IBS-D with Bifidobacterium longum, Bifidobacterium bifidum, Bifidobacterium lactis, Bifidobacterium infantis, and Lactobacillus acidophilus resulted in a change in inflammation-related metabolites (27). Individuals with IBS on a gluten-free diet with probiotic supplementation of *Lactobacillus* and *Bifidobacterium* spp. saw an overall improvement in symptoms (28).

Here, we present a large-scale metagenomic study to characterize and compare the microbiome compositions and functional potentials of controls and individuals with IBS at baseline, as well as the microbiome changes associated with 4 months of daily synbiotic administration to subjects with IBS. Our primary goals were to (i) identify microbiome features associated with IBS and (ii) investigate whether synbiotics alter these IBS-associated microbiome features. We hypothesized that metagenomic features distinguish healthy from IBS microbiome subtypes and that daily synbiotic supplementation modulates the microbiomes of the individuals with IBS.

## RESULTS

### IBS and healthy subject demographics.

We included a total of 612 subjects in this study. All participants were geographically distributed across the United States. Subjects self-reported as healthy with no comorbidities were included as the healthy control population. There were 490 subjects with IBS and 122 subjects in the healthy control population (Table 1). The average age of each cohort was between 40 and 47 years, and while the healthy population was matched based on sex, the IBS population was ∼65% female. A proportion of each population had undefined sex. Of the 490 IBS subjects, 134 subjects had at least 2 time points, 56 subjects had 3 time points, 28 subjects had 4 time points, 15 subjects had 5 time points, 5 subjects had 6 time points, and 1 subject had 7 time points. Female subjects were the predominant population of each IBS subtype, with approximately 68% occurrence with IBS-C, 58% with IBS-D, 66% with IBS-A, and 49% with IBS-U.

**TABLE 1:** Subject demographics^*a*^

Phenotype	No. of subjects			
	Total	Female (age [yr] ± SD)	Male (age [yr] ± SD)	Unspecified (age [yr] ± SD)
Healthy	122	54 (44 ± 13)	52 (44 ± 12.6)	16 (41.9 ± 9.2)
IBS (total)	490	301 (46.5 ± 15.5)	158 (41.6 ± 15.3)	31 (43.3 ± 16.9)
IBS-C (constipation)	185	126 (45.5 ± 14.9)	50 (37.4 ± 13.5)	9 (41.7 ± 13.1)
IBS-D (diarrhea)	86	50 (41.9 ± 14.5)	32 (44.3 ± 16.5)	4 (40.7 ± 12.1)
IBS-A (alternating)	88	58 (45.4 ± 15.6)	26 (37.6 ± 12.4)	4 (41.5 ± 14)
IBS-U (unspecified)	131	64 (53.6 ± 15.6)	49 (46.5 ± 16.3)	18 (45.6 ± 21.5)

a “Healthy” controls are self-reported as healthy subjects with no existing comorbidities. Subjects with IBS are also self-reported. The subtype designation is based on the subject symptoms. For the alternating designation, subjects experienced symptoms of constipation and diarrhea. Cohort populations are further classified by gender. Average and standard deviation for age groups are listed next to each population.

### Reduced microbial diversity and microbial signatures associated with IBS.

**FIG 1:**
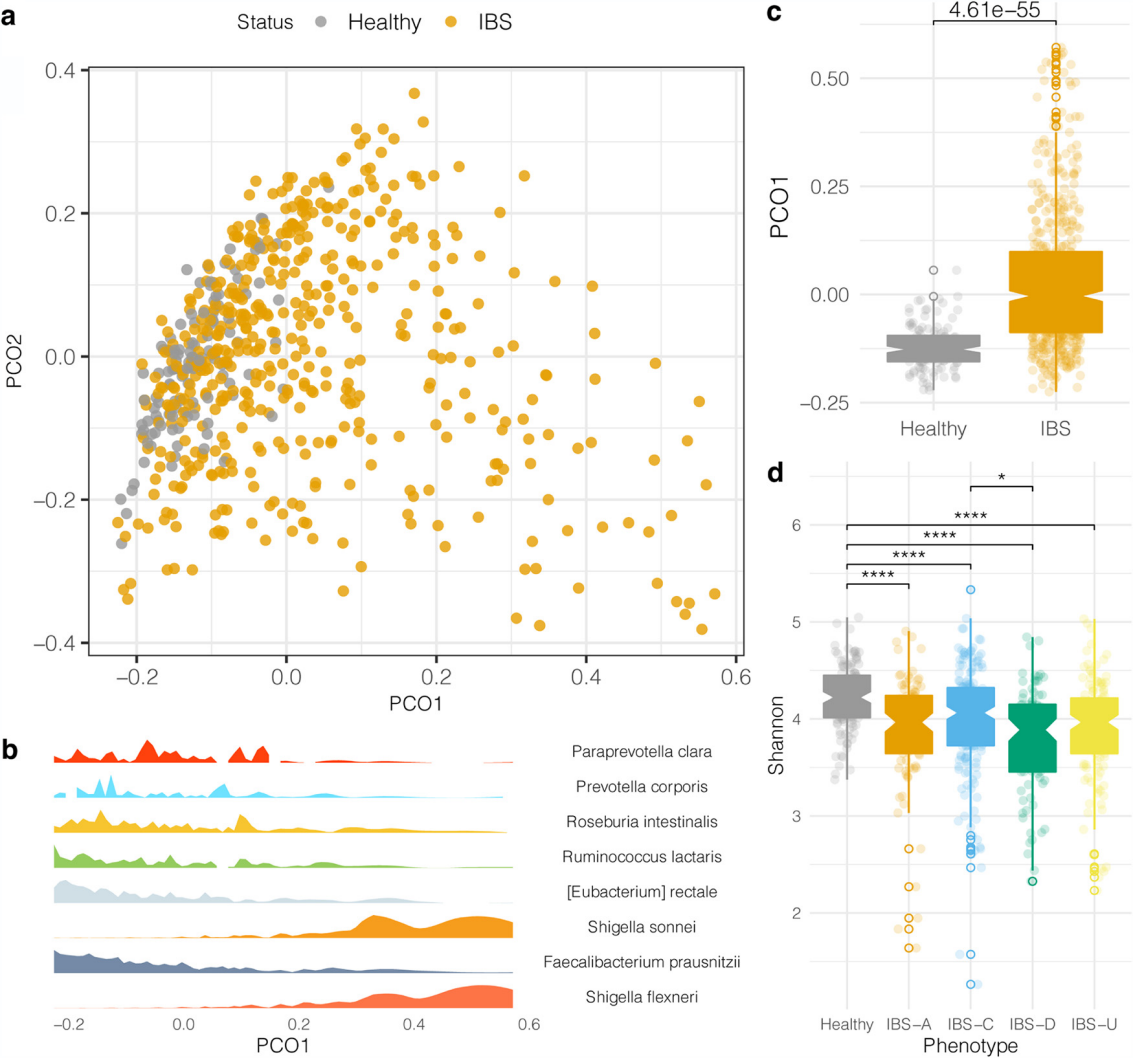
Microbiome profiles of the healthy and IBS cohorts. (a) Principal-coordinate analysis based on the Bray-Curtis dissimilarity distance matrix of the IBS and healthy microbiomes. (b) A Random Forest analysis was employed to differentiate microbes between healthy and IBS subtypes. The density of microbes selected from Random Forest analysis corresponds to the sample distribution along the PCO1 axis. (c) Boxplot of the microbiome distributions along the PCO1 axis. An unpaired *t* test was computed. Open circle data points are outliers outside 1.5 times the interquartile range. (d) Shannon index between healthy and each IBS subtype cohort. Open-circle data points are outliers outside 1.5 times the interquartile range. Unpaired *t* tests were conducted, and *P* values were adjusted with Benjamini-Hochberg false-discovery rate (FDR) for multiple comparisons. *, *P* < 0.05; ****, *P* < 0.0001.

First, to compare the microbial community compositions between the IBS and healthy control populations, a principal-coordinate analysis was performed to visualize the beta diversity between the two cohorts (Fig. 1a). Principal coordinate 1 (PCO1) was significantly different between IBS and healthy groups (Fig. 1c). Random Forest analysis was used to identify microbial taxonomic features that were predictive of IBS, which demonstrated that IBS differences across PCO1 were driven by an increased relative abundance of *Enterobacterales* species and reduction in Eubacterium rectale and Faecalibacterium prausnitzii compared to healthy samples (Fig. 1b). Next, when calculating alpha diversity metrics, there was a significant reduction in the Shannon index in IBS subtypes compared to the healthy control population (Fig. 1d).

**FIG 2:**
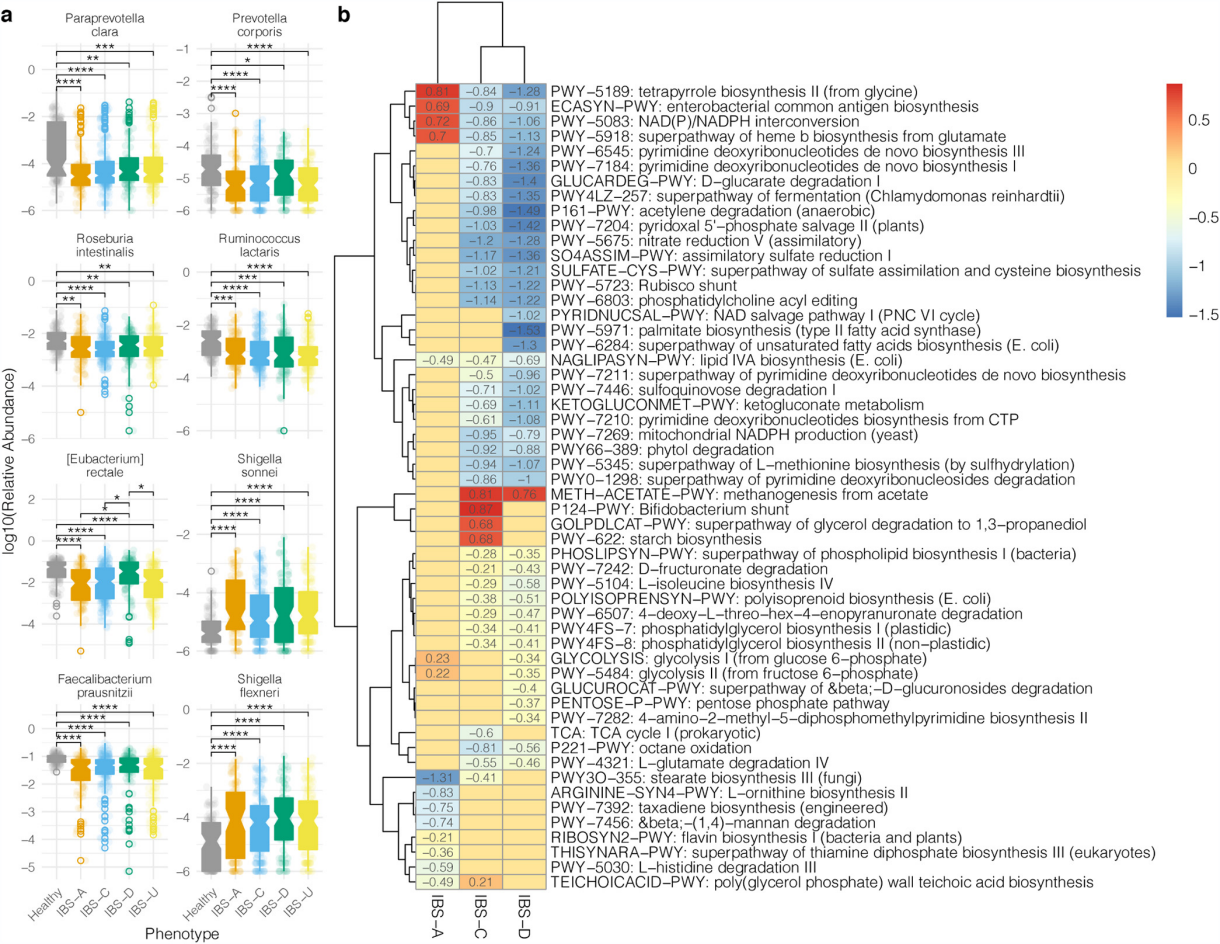
Microbes and pathways that differentiate healthy and IBS cohorts. (a) Log base 10 of relative abundances of the microbes associated with healthy and IBS populations. A Random Forest analysis was used to determine microbes that contribute to differentiating healthy and IBS subjects. The relative abundances of a subset of the microbes were plotted for healthy subjects and each IBS subtype. Open-circle data points are outliers outside 1.5 times the interquartile range. *t* tests were calculated. *P* values were adjusted for multiple-comparison testing by false-discovery rate corrections. Nonsignificant comparisons were omitted. (b) Functional pathways associated with healthy and IBS gut microbiomes. Multivariate linear association testing with Maaslin2 was used to determine pathways associated with IBS relative to the healthy control population. Values indicate the beta coefficient from linear association testing. Pathways listed were filtered based on a *q* value of <0.1 and beta coefficients of >0.2 or <−0.2. *, *P* < 0.05; **, *P* < 0.01; ***, *P* < 0.001; ****, *P* < 0.0001.

Based on whole-genome shotgun metagenomic sequencing, microbial signatures distinguish the healthy control and IBS populations. Using a permutated multivariate analysis of variance, we calculated a significant variation that explained the difference between the microbiomes of healthy and IBS subtypes (*R*^2^ = 0.028, *P* < 0.001). We performed a Random Forest analysis to identify the distinguishing microbes between healthy and IBS phenotypes. To identify statistically significant changes in the relative abundances of microbes within healthy or IBS subtypes, we performed an unpaired *t* test and adjusted *P* values for multiple testing corrections. This analysis revealed Eubacterium rectale and Faecalibacterium prausnitzii as significantly increased microbial species in the healthy control population relative to all IBS subtypes (Fig. 2), while we found species of *Shigella* elevated in IBS (Fig. 2). We further interrogated the microbial differences between IBS subtypes and found that Paraprevotella clara, Prevotella corporis, Roseburia intestinalis, and Ruminococcus lactaris significantly decreased in relative abundance in different IBS subtypes relative to the healthy control population (Fig. 2).

### Functional profile of the gut microbiome associated with IBS and healthy subjects.

To determine the functional profiles of the gut microbiome associated with IBS, we mapped the metagenomic reads against the MetaCyc database with Humann3 to identify pathway abundances. We detected a total of 471 pathways across all metagenomes. Multivariate linear association testing identified pathways associated with each IBS dominant subtype relative to the healthy control cohort (Fig. 2). Pathways involved in tetrapyrrole biosynthesis from glycine, enterobacterial common antigen biosynthesis, NADP/NADPH interconversion, and the superpathway of heme b biosynthesis from glutamate were positively associated with IBS-A (Fig. 2). Methanogenesis from acetate was associated with IBS-C and IBS-D (Fig. 2). Pathways involved in the *Bifidobacterium* shunt, the superpathway of glycerol degradation to 1,3-propanediol, and starch biosynthesis were associated with IBS-C (Fig. 2). Meanwhile, pathways associated with amino acid and ribonucleotide biosynthesis, polysaccharide degradation, and fermentation were associated with healthy microbiome functional profiles (Fig. 2).

### Probiotics increase in relative abundance in gut microbiome of subjects with IBS.

**FIG 3:**
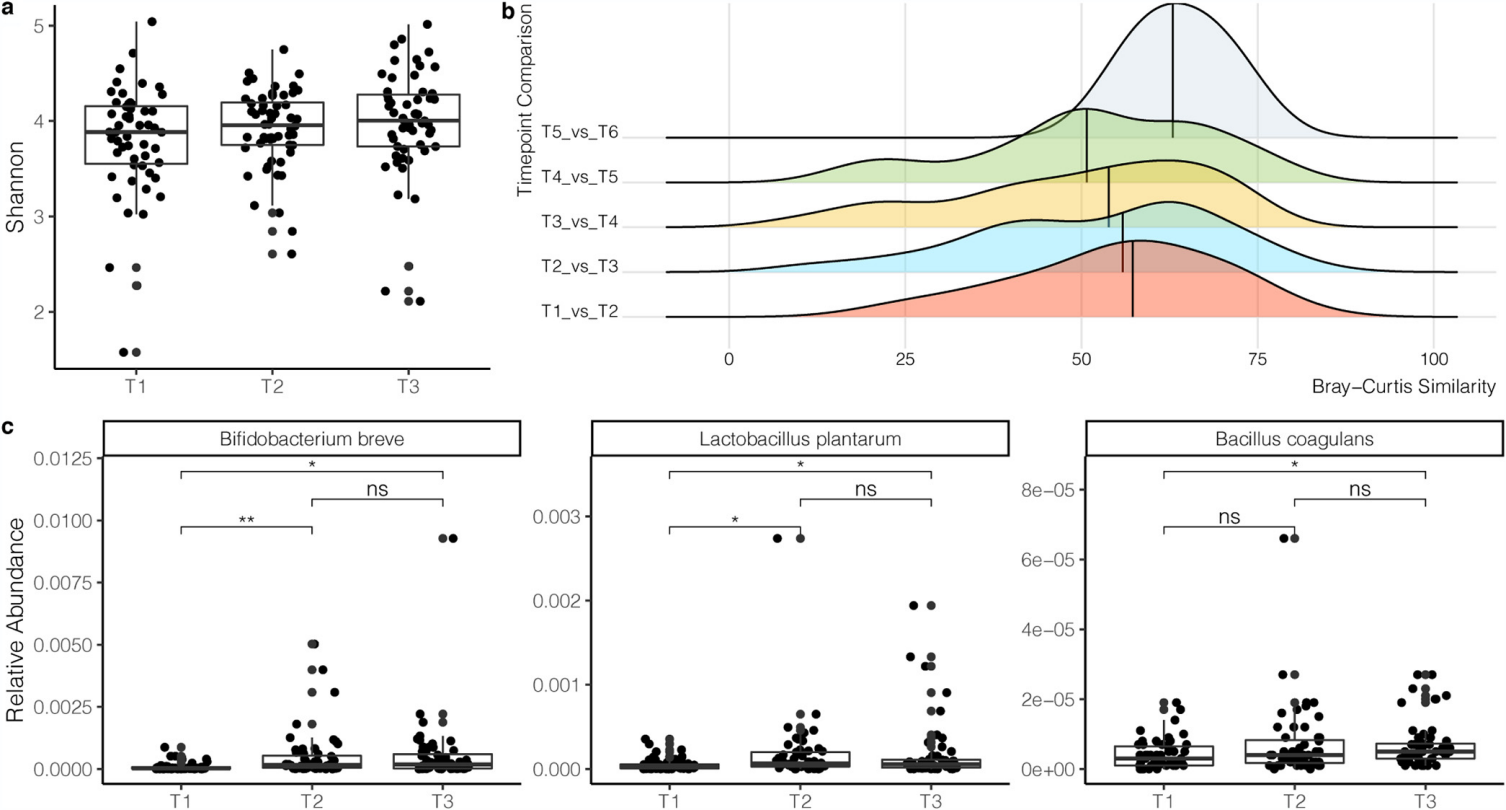
Longitudinal microbiome diversity and relative abundances of probiotics in subjects with IBS. (a) Shannon index of the microbiome composition from subjects with time points 1 to 3. (b) Bray-Curtis similarity of time points within each individual. Each time point is compared to each subsequent time point. (c) Relative abundances of probiotic species detected in the gut microbiome of subjects across 3 time points. *t* tests were computed with false-discovery rate (FDR)-adjusted *P* values. *, *P* value < 0.05; **, *P* value < 0.01; ****, *P* value < 0.0001; ns, not significant.

Within a subset of the IBS population, there were 134 individuals with at least two time points and 56 individuals with three time points. The average number of days between time points 1 and 2 was 154.8 ± 80.5 (standard deviation [SD]) days, and that between time points 2 and 3 was 194.9 ± 144.5 (SD) days. To investigate whether there were changes in alpha diversity across time, we performed a linear mixed-effects model to control for the effect from the individual. Based on the calculations on the longitudinal data set controlling for the individual, there were no significant increases in the Shannon index, richness, or evenness. Alpha diversity did increase between time points 1 and 3, although this was not significant (Fig. 3). Next, we calculated the Bray-Curtis similarity (Bray-Curtis similarity = 1 – Bray-Curtis dissimilarity) of microbiome composition to investigate changes in the microbiome across time. There was no significant difference from one time point to the next (Fig. 3) or when comparing the first time point with each subsequent time point (data not shown). However, there was a shift in the median toward lower Bray-Curtis similarity indices across longitudinal time points 1 to 5 (Fig. 3). A permutated multivariate analysis of variance was performed across all time points to calculate microbiome variance across longitudinal samples. There was a significant difference between all longitudinal samples from time point 1 and time point 3 (*R*^2^ = 0.0088, *P* = 0.035). The average time between time points 1 and 3 was 335.9 ± 170.5 (SD) days.

Given the heterogeneity of the symptoms experienced by the IBS population, there was no one common formula or probiotic for any of the dominant subtypes. Each individually formulated synbiotic formulation contained approximately 4 to 8 probiotic strains and 1 to 2 prebiotics, each at different concentrations. The most common probiotics formulated for IBS-A were Bifidobacterium longum, Bifidobacterium breve, and Saccharomyces boulardii in 47%, 43%, and 32% of the formulations, respectively. For IBS-C, B. longum, *B. breve*, and Lactobacillus plantarum were included in 85%, 50%, and 41% of the formulations, respectively. For IBS-D, *B. breve*, *S. boulardii*, and turmeric powder (prebiotic) were included in 47%, 29%, and 25% of formulations, respectively. For IBS-U, *B. breve*, *L. plantarum*, and *S. boulardii* were included in 64%, 31%, and 31% of formulations, respectively. Bacillus coagulans was included in a proportion of each IBS subtype formulation at a frequency of 20 to 30%.

We investigated whether the probiotics we provided were detected at an increased abundance level in the subject gut microbiomes across time. In the longitudinal data set, *B. breve*, *L. plantarum*, and *B. coagulans* significantly increased in abundance across time (Fig. 3). *B. breve* significantly increased from time point 1 to time points 2 and 3, but there was no significant change between the second and third time points (Fig. 3). *L. plantarum* was significantly increased in abundance at time points 2 and 3 compared to time point 1 (Fig. 3). *B. coagulans* significantly increased in abundance between time points 1 and 3 (Fig. 3). There was not a significant increase in the relative abundance of B. longum across time points 1 to 3 (data not shown).

### Microbiome features associated with each IBS subtype.

**FIG 4:**
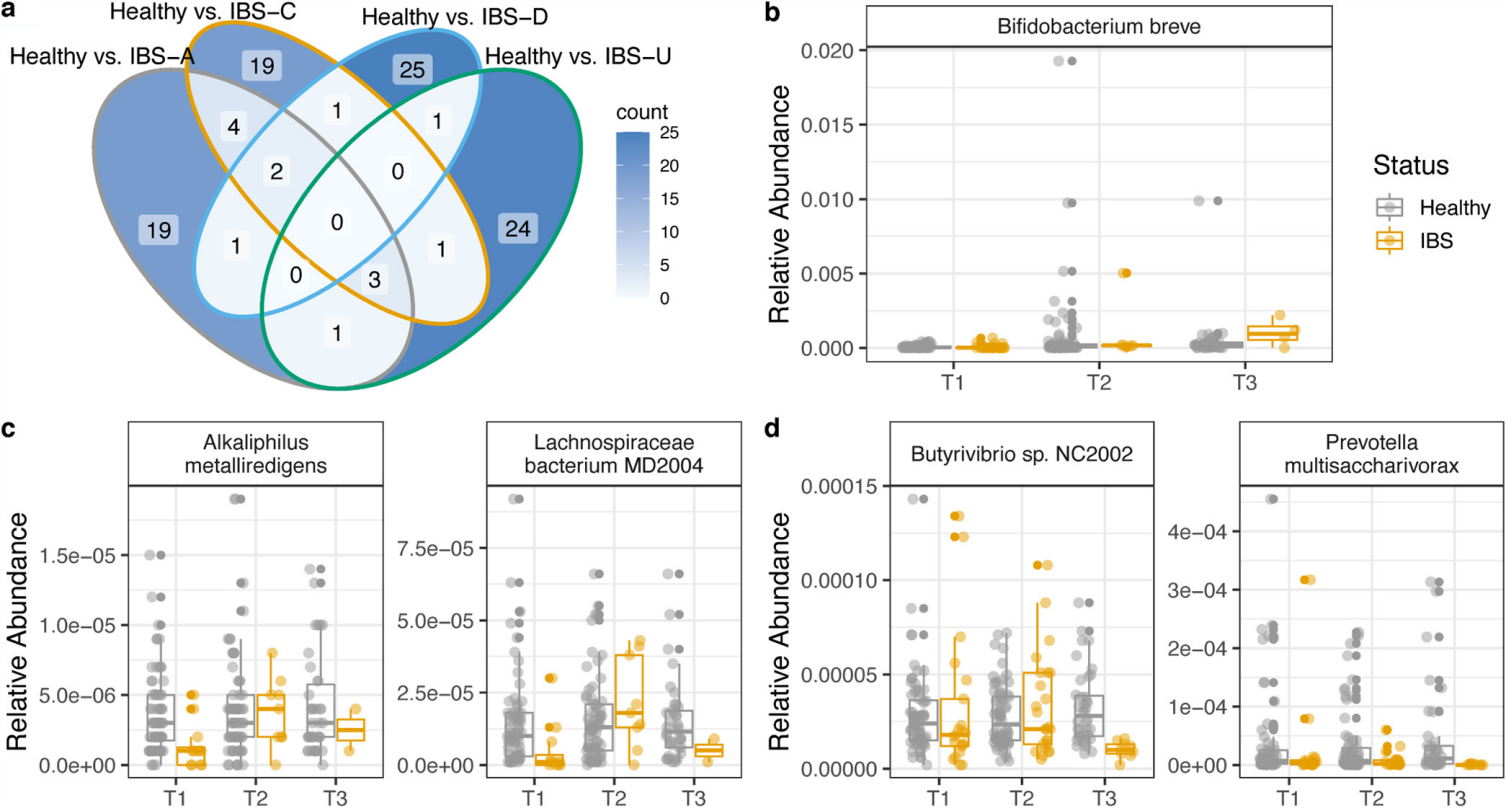
Microbial features associated with each IBS subtype. (a) Venn diagram of unique and shared microbes from Random Forest analysis comparing healthy subjects and each IBS subtype. (b to d) The relative abundances of microbes across longitudinal healthy subjects and IBS subtypes. Microbes were determined by Random Forest analysis between healthy subjects and IBS subtype and selected based on a significant change across IBS subtype time points. Microbes with significant change across time in IBS-A (b), IBS-D (c), and IBS-U (d) cohorts.

**FIG 5:**
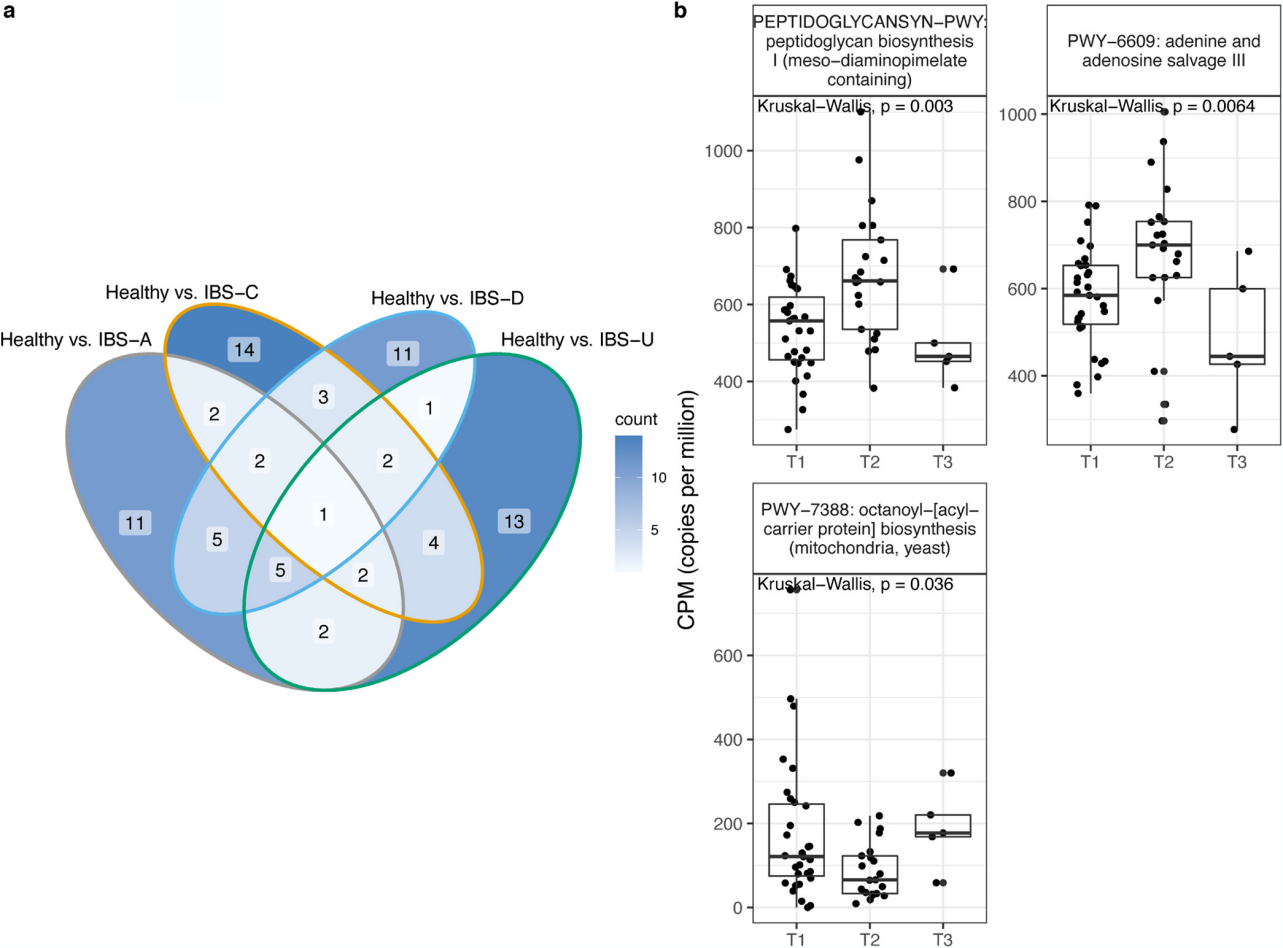
Microbial features associated with each IBS subtype. (a) Venn diagram of unique and shared pathways from Random Forest analysis comparing healthy subjects and each IBS subtype. (b) The copies per million of pathways across longitudinal IBS-C subtypes determined by Random Forest analysis.

To determine microbiome composition and pathway features that were associated with each subtype, we performed a Random Forest analysis comparing each IBS subtype to the healthy control population. Of the top 30 features from each analysis, there were few overlapping features between each subtype comparison, and the majority of features were unique to each IBS subtype compared to healthy controls. Microbial composition and pathways that distinguish healthy and IBS subtypes are listed in Tables S1 and S2 in the supplemental material. With longitudinal analysis, we found that there were significant changes in the relative abundances of microbes from IBS subtypes determined by Random Forest analysis (Fig. 4). Alkaliphilus metalliredigens and *Lachnospiracea*e bacterium MD2004 significantly changed across time in the longitudinal IBS-D cohort (Kruskal-Wallis, *P* = 0.049 and 0.028, respectively). *Butyrivibrio* and Prevotella multisaccharivorax significantly changed across time in the longitudinal IBS-U cohort (Kruskal-Wallis, *P* = 0.024 and 0.0086, respectively). Pathways of peptidoglycan synthesis, adenine and adenosine salvage, and octanoyl-[acyl-carrier protein] biosynthesis significantly changed across time in IBS-C (Fig. 5).

10.1128/mSystems.01215-21.1 TABLE S1 Microbes that distinguish each IBS subtype from healthy controls. A Random Forest analysis was performed to identify features that distinguish each IBS subtype from healthy controls. Unique and shared features selected from the top 30 mean decrease GINI values from each analysis are listed. Download Table S1, CSV file, 0.00 MB. Copyright © 2021 Phan et al. 2021 Phan et al. https://creativecommons.org/licenses/by/4.0/ This content is distributed under the terms of the Creative Commons Attribution 4.0 International license.

10.1128/mSystems.01215-21.2 TABLE S2 Microbial pathways that distinguish each IBS subtype from healthy controls. A Random Forest analysis was performed to identify features that distinguish each IBS subtype from healthy controls. Unique and shared features selected from the top 30 mean decrease GINI values from each analysis are listed. Download Table S2, CSV file, 0.01 MB. Copyright © 2021 Phan et al. 2021 Phan et al. https://creativecommons.org/licenses/by/4.0/ This content is distributed under the terms of the Creative Commons Attribution 4.0 International license.

## DISCUSSION

Although IBS is prevalent across the population, the underlying factors contributing to the syndrome make diagnosis and treatment challenging to define and standardize. Previous amplicon-based studies have identified changes in microbiome composition and diversity in individuals with IBS compared to a healthy control population (29, 30). Concomitant with previous findings, our study corroborates the significant microbial community composition differences and diversity between healthy individuals and people with IBS. Unlike other studies, whole-metagenome shotgun sequencing enabled us to identify species and metabolic pathways associated with the dominant subtypes of IBS. In addition, our precision probiotics for individuals with IBS showed an increased relative abundance of probiotics detected in the gut microbiome across time. Of subjects with three time points, 91% had all three of the common probiotic species we included in formulations. Clinical studies that administer probiotics to individuals with IBS have shown reduced symptom severity and gut discomfort (25, 27, 28). Although we did not find a significant change in alpha diversity or reduction of *Shigella* spp. in the longitudinal IBS profiles with probiotic supplementation, there was a significant change in microbial species and pathways across time in IBS subtypes. Further research is needed to assess longitudinal changes in microbiome function in response to probiotics in IBS.

Individuals with IBS demonstrated a significant reduction in alpha diversity and predicted anti-inflammatory bacteria and a concomitant increased proportion of predicted proinflammatory bacteria, such as *Shigella*. The reduction in alpha diversity may be due to medications such as antibiotics or overgrowth of specific bacteria (e.g., reference 31). However, there was no significant difference in the beta diversity in the microbiomes of subjects who have or have not taken antibiotics within the last 3 months of their stool sample collection. Consistent with IBS-A, IBS-C, and Crohn’s disease studies, we found lower relative abundances of the anti-inflammatory microbe *F. prausnitzii* in individuals with IBS than in the healthy cohort (29, 32–35). In contrast to previous amplicon-based studies that did not find a reduced abundance of *F. prausnitzii* in IBS-D (35–37), we detected *F. prausnitzii* significantly reduced in IBS-D compared to controls. *F. prausnitzii* enhances gut barrier protection and produces butyrate, a short-chain fatty acid (SCFA) essential for gut health (29, 32, 38, 39). Roseburia intestinalis has an anti-inflammatory role in the gut and is reduced in individuals with Crohn’s disease (40, 41). *R. intestinalis* was significantly reduced in IBS-C and IBS-D subtype (Fig. 2). *Shigella* spp., major contributors to diarrheal disease (42) and associated with postinfectious IBS (43), were found to be increased in the IBS subtypes (Fig. 2).

The other differentially abundant microbes have an unclear role in IBS. Ruminococcus lactaris is negatively correlated with interleukin-8 (IL-8) (44) and is more abundant in a non-chronic kidney disease cohort (45) but has also been shown to be associated with a high-fat diet in a murine diabetes model (46). Eubacterium rectale is a butyrate producer associated with infant gut microbiome development (47) but is also associated with obesity and dysbiosis (48). In a recent metagenomic assembly study of E. rectale, there were different subspecies due to genetic and geographic dispersal in human populations, revealing differences in subspecies physiologies and metabolisms (49). *Prevotella* spp. are common in non-Western plant-rich diets (50) and decreased in individuals with constipation (51) but have also been associated with chronic inflammatory conditions (52, 53). These studies indicate that the role of some microbes detected in this study is context and environment dependent.

Functional analysis identified pathways associated with each of the phenotypic classifications of IBS. The methanogenesis from an acetate pathway was associated with IBS-C (Fig. 2). Methanogenesis contributes to methane production, which is correlated with the severity of constipation (54) and may be useful as a diagnostic indicator of constipation-predominant IBS (55, 56). Surprisingly, methanogenesis was also associated with IBS-D. Previous studies have demonstrated the reduction of methanogens in IBS-D (57). The *Bifidobacterium* shunt was also associated with IBS-C. The *Bifidobacterium* shunt, also called the fructose-6-phosphate shunt, produces short-chain fatty acids (SCFAs) and other organic compounds (58, 59). An overabundance of short-chain fatty acids, substrates for methanogenesis, may lead to gut symptoms in IBS. Depending on the chemical and microbial microenvironment, SCFAs can regulate the growth and virulence of enteric pathogens (60). In addition, SCFA stimulates water absorption in the colon (61). If too much water is absorbed, the stool becomes more solid, resulting in constipation. Thus, factors affecting host physiology in IBS may depend upon the microenvironments and microbes present in the gut. These findings suggest that future work should focus on formulating synbiotics that may reduce methanogenesis or regulate the production of SCFAs to improve IBS symptoms.

The enterobacterial common antigen (ECA) biosynthesis pathway was associated with IBS-A. The ECA is one of the components of the outer membrane of Gram-negative bacteria, and its association with IBS-A may indicate the increased presence of *Enterobacterales* in the gut microbiome. Interestingly, the ECA may contribute to virulence and protect enteric pathogens from bile salts and antibiotics (62–64). Bile acids protect the host from infection, contributing to overall gut intestinal health (65). ECA protection against bile acids and antibiotics may make IBS-A challenging to treat with antibiotics and may contribute to dysbiosis. These results suggest that common antibiotic treatments for IBS may not be ideal for alleviating symptoms or treating the possible underlying microbiome triggers associated with IBS-A.

IBS is heterogeneous; a universal cocktail of probiotics may not comprehensively target all symptoms experienced by individuals with IBS. Therefore, individually formulated prebiotics and probiotics may be able to address the more common symptoms experienced by individuals with IBS. There were common strains included in formulas to specifically target constipation and diarrhea. Bifidobacterium longum was included in formulations for constipation because studies have demonstrated treatment efficacy in stool frequency and consistency (66, 67). *B. breve* was included in formulations for diarrhea because it has been demonstrated to reduce severity and incidence of diarrhea (68, 69). Although B. longum did not increase in relative abundance across time, the presence of B. longum may still promote gut health through cross-feeding mechanisms that lead to the production of short-chain fatty acids (70, 71). Further investigation is needed to identify potential functional changes in microbiome metabolism with daily probiotic supplementation in IBS and whether symptoms associated with IBS can be improved.

There are several limitations to this current study. First, the self-reporting nature of IBS is a limitation to this study. For official diagnosis of IBS, the Rome IV criteria assess symptoms related to stool consistency and appearance, recurrent abdominal pain, and changes in bowel habits (72, 73). Although the health and diet questionnaire included questions regarding gut symptoms and chronic conditions, a formal diagnosis was not verified. For potential lifestyle modifications in addition to probiotic supplementation, diet changes may also be an important factor in alleviating symptoms or changing the microbiome (74–76). Low fermentable oligosaccharides, disaccharides, monosaccharides, and polyols (FODMAP) diet (LFD) and low-lactose diet (LLD) have been shown to reduce the IBS symptom severity score (IBS-SSS), and subjects on LFD had significantly less abdominal pain, bloating, and gas production (75). These diet interventions were not accessed in this study. Second, this study was not designed to investigate longitudinal assessments of comprehensive gut issues experienced by the individuals with IBS. This hindered us in identifying whether gut symptoms were alleviated by daily probiotic supplementation or whether there were associations with certain probiotic formulations in improving certain symptoms in IBS. However, because the relative abundances of the common probiotics formulated for constipation and diarrhea were increased across time, these results may inform future studies. Additional research is also needed to determine the roles of specific pathways in the etiology of IBS.

In summary, we reported differentially abundant microbes and functional pathways associated with IBS and each IBS subtype relative to healthy controls. Of the microbes and pathways associated with each subtype, a subset was significantly changed in relative abundance across time in the IBS subtype populations. We also identified an increased relative abundance of probiotics in the gut microbiomes of people with IBS across time. These data may help inform future studies and therapeutic strategies by identifying important microbes and pathways associated with each IBS subtype. Probiotic strains or prebiotic ingredients can be formulated to target specific pathways or microbes that may be contributing to symptoms. Without these analyses, a blanket treatment may not resolve issues experienced by individuals with IBS. As IBS is a multifactorial syndrome, there is no one-size-fits-all approach to target all symptoms experienced by individuals with IBS. A combination of diet and probiotics may be needed to alleviate symptoms of IBS. Longitudinal monitoring of the gut microbiome is also important to understand changes associated with symptom progression. Further research is needed to identify the pathway benefits and interactions of prebiotic and probiotic supplementation with gut health and influence on IBS symptoms.

## MATERIALS AND METHODS

### Participants and sample collection.

Users of our (Sun Genomics, San Diego, CA) gut microbiome test kit (Floré gut health test kit) submitted a stool sample for metagenomic sequencing. The stool sample was collected by the user with provided gut testing kit instructions. Samples were collected in accordance with IRB no. SG-04142018-001 with informed consent form 001-B. A sterile swab was used for the first collection tube to collect and store the stool sample in a stabilization buffer. The second sample was collected via the Easi-Collect component (GE). Samples were mailed via FedEx to the Floré lab for analysis.

A total of 612 participants were included in this study (Table 1). All participants completed a health and diet survey that asked questions about health status and dietary preferences. The control population included in this study was self-reported as healthy with no listed comorbidities with a body mass index (BMI) range from 18.5 to 25 (Table 1) (77). The IBS population was also self-reported and included the symptoms associated with the syndrome, including constipation, diarrhea, a mix of both constipation and diarrhea, or unspecified.

In addition, longitudinal samples from IBS subjects were assessed to identify specific microbiome changes during the course of prebiotic and probiotic supplementation. Each formulation includes 4 to 8 probiotic strains and 1 to 2 prebiotics, each at different concentrations from a biobank of over 100 possible ingredients supported by the clinical literature. Longitudinal time points were approximately 5 months apart with 4 months of probiotic and prebiotic supplementation.

### Metagenomic sequencing and analysis.

For DNA extractions, samples were first processed with a tissue homogenizer and then lysed with a lysis buffer and proteinase K. DNA was extracted and purified with a proprietary method (patents 10428370 and 10837046 [78, 79]). Library preparation was performed with DNA sonication, end-repair, and adaptor ligation with NEBNext reagents. Size selection was performed with MagJet magnetic beads according to the manufacturer’s instructions. Library quantitation was performed with quantitative PCR (qPCR), and sequencing was performed with an Illumina NextSeq 550 (Illumina, San Diego, CA). After sequencing, reads were quality filtered and processed. Metagenomic reads were decontaminated from human reads using Bowtie2. After decontamination, there was an average of 6,581,844 reads per sample (SD = 4,426,117) with a minimum of 1 million reads to be included in downstream analyses. Next, reads were aligned to a hand-curated database of over 23,000 species. Humann3 was used for pathway analysis (80). Pathway abundance was normalized to copies per million (cpm).

### Statistical analyses.

All statistical analyses were performed in R. Principal-coordinate analysis was performed with a Bray-Curtis dissimilarity matrix to compare between-sample diversities. Within-sample diversity was calculated with the Shannon diversity index. To calculate variance between samples based on metadata classifications, permutational multivariate analysis of variance (PERMANOVA) was performed with the “adonis” function from the “vegan” package (81). Specifically, the influence of health status was computed across the microbiome composition and pathway abundance profiles. MaasLin2 was used for distinguishing pathway features between healthy and IBS subtypes (82).
